# DNA Methylation Analysis in Plasma Cell-Free DNA and Paired CTCs of NSCLC Patients before and after Osimertinib Treatment

**DOI:** 10.3390/cancers13235974

**Published:** 2021-11-27

**Authors:** Aliki Ntzifa, Dora Londra, Theodoros Rampias, Athanasios Kotsakis, Vassilis Georgoulias, Evi Lianidou

**Affiliations:** 1 Analysis of Circulating Tumor Cells Lab, Lab of Analytical Chemistry, Department of Chemistry, National and Kapodistrian University of Athens, 15771 Athens, Greece; alntzi@chem.uoa.gr (A.N.); doralo@chem.uoa.gr (D.L.); 2 Basic Research Center, Biomedical Research Foundation of the Academy of Athens, 11527 Athens, Greece; trampias@bioacademy.gr; 3 Faculty of Medicine, School of Health Sciences, University of Thessaly, 41110 Larissa, Greece; thankotsakis@uth.gr; 4 Department of Medical Oncology, Hellenic Oncology Research Group (HORG), 11471 Athens, Greece; georgulv@otenet.gr

**Keywords:** DNA methylation, liquid biopsy, circulating tumor cells, cell-free DNA, NSCLC, osimertinib, real-time MSP

## Abstract

**Simple Summary:**

Liquid biopsy is a highly useful tool for the management of NSCLC patients and could provide valuable information on early detection of resistance to osimertinib. Epigenetic biomarkers are very promising for the early diagnosis, prognosis, and prediction of drug response in many types of cancer. We performed a DNA methylation analysis in plasma cell-free DNA and paired CTCs of NSCLC patients before osimertinib treatment and at progression of disease (PD). Our results revealed a significant increase in DNA methylation at PD. Epigenetic alterations should be further evaluated as a possible resistance mechanism to osimertinib and their detection in liquid biopsy samples can be valuable for the follow-up of patients in real time.

**Abstract:**

Osimertinib has been an effective second-line treatment in EGFR mutant NSCLC patients; however, resistance inevitably occurs. DNA methylation has been previously implicated in NSCLC progression and often in therapy resistance, however its distinct role in osimertinib resistance is not elucidated as yet. In the present study, we directly compared DNA methylation of nine selected genes (*RASSF1A*, *RASSF10*, *APC*, *WIF-1*, *BRMS1*, *SLFN11*, *RARβ*, *SHISA3*, and *FOXA1*) in plasma-cfDNA and paired CTCs of NSCLC patients who were longitudinally monitored during osimertinib treatment. Peripheral blood (PB) from 42 NSCLC patients was obtained at two time points: (a) baseline: before treatment with osimertinib and (b) at progression of disease (PD). DNA methylation of the selected genes was detected in plasma-cfDNA (*n* = 80) and in paired CTCs (*n* = 74). Direct comparison of DNA methylation of six genes between plasma-cfDNA and paired CTC samples (*n* = 70) revealed a low concordance, indicating that CTCs and cfDNA give complementary information. DNA methylation analysis of plasma-cfDNA and CTCs indicated that when at least one of these genes was methylated there was a statistically significant increase at PD compared to baseline (*p* = 0.031). For the first time, DNA methylation analysis in plasma-cfDNA and paired CTCs of NSCLC patients during osimertinib therapy indicated that DNA methylation of these genes could be a possible resistance mechanism.

## 1. Introduction

The therapeutic landscape of non-small cell lung cancer (NSCLC) patients that carry somatic mutations in the tyrosine kinase (TK) domain of epidermal growth factor receptor (EGFR) has been successfully validated during the last two decades and the prolonged survival of those patients who benefit from EGFR TKIs has already proven through numerous clinical studies [1,2,3,4]. Osimertinib, a third generation EGFR TKI, is successfully administered both as first [5] and as second line treatment [6,7] in EGFR mutant NSCLC patients. However, in most cases, resistance arises inevitably and the identification of the molecular mechanisms that lead to the progression of disease (PD) is crucial for the appropriate sequential treatment. Besides, the extensive heterogeneous nature of NSCLC further complicates the identification of the resistance mechanisms that occur at progression of disease. EGFR dependent mechanisms are well defined and easy to target but there is still a big percentage of molecular alterations responsible for treatment inefficiency that remains unexplored so far [8,9,10].

Epigenetic regulation of important cell cycle pathways such as cell cycle control, proliferation, apoptosis, cellular adhesion, motility, and DNA repair is often responsible for lung cancer initiation and progression [11]. DNA methylation as part of these modifications has been an important mechanism especially when it occurs in CpG rich regions of the 5′ ends of many genes. This is a common epigenetic process that leads to the silencing of tumor suppressor genes and there is evidence that this can either act as a sole inactivating mechanism or in combination with the presence of mutations [12,13]. In NSCLC, various studies have shown the prognostic and diagnostic significance of DNA methylation of such tumor suppressor genes [14,15]. Moreover, there is accumulating evidence that the dominance of tumor cancer cells undergo epigenetic modifications and their transition to a more drug tolerant cell subpopulation leads to the rapid acquisition of treatment resistance [16].

Liquid biopsy has drawn major attention in the last few years as a highly useful tool for the management of cancer patients and has already shown its clinical impact on the early detection, minimal residual disease and tracking of treatment resistance [17,18]. The identification of circulating epigenetic biomarkers through DNA methylation studies is of utmost clinical importance in the liquid biopsy field and can be used for the diagnosis, prognosis, and prediction of drug response [19,20]. Notwithstanding that mutation analysis in liquid biopsy is already established through its clinical utility, it cannot always provide consistent results due to tumoral heterogeneity. Therefore, epigenetic analysis of cfDNA could serve as a promising alternative and broaden the spectrum of diagnostic applications, since reversible epigenetic aberrations are tumor-specific and reflect disease progression [21,22]. A representative example of its potential diagnostic utility is the latest work of Liu et al. where cfDNA bisulfite sequencing revealed distinct methylation patterns among more than 50 cancer types across all stages [23]. Furthermore, another study has shown that lung cancer subtyping based on cfDNA methylation analysis of *APC*, *HOXA9*, *RARβ2*, and *RASSF1A* could be critical for the accurate diagnosis of patients and for their prognosis through an optimal selection of treatment management [24]. Epigenetic alterations in circulating tumor cells (CTCs) were first described in 2011 [25] but these studies are still limited to a few cancer types [26,27,28]. However, they are quite informative about CTC dissemination, metastatic tumor formation and progression [29,30]. Recently, a unique CTC DNA methylation signature of lung cancer patients, distinct from the primary tumor, revealed a stemness feature during metastasis [31].

In the present study, we directly compared the DNA methylation of nine selected genes in plasma-cfDNA and paired CTCs of NSCLC patients before and after osimertinib therapy, namely RAS-association domain family 1 isoform A (*RASSF1A*), Ras-association domain family 10 (*RASSF10*), Wnt inhibitory factor-1 (*WIF-1*), Adenomatous polyposis coli (*APC*), Breast cancer metastasis suppressor 1 (*BRMS1*), DNA/RNA helicase Schlafen-11 (*SLFN11*), *SHISA3*, retinoic acid receptor-beta (*RARβ*) and forkhead box protein A1 (*FOXA1*). These genes were selected based on previous publications suggesting being epigenetically silenced in lung cancer, and on a meta-analysis carried out on lung adenocarcinoma methylation and transcriptomic data from the Cancer Genome Atlas (TCGA).

Epigenetic inactivation of several RASSF members has already been reported in lung cancer. *RASSF1A* action stimulates mitotic arrest, DNA repair and apoptosis, and controls the cell cycle and cell migration [32]. On the other hand, the epigenetic status of *RASSF10* is not fully elucidated in lung cancer and only few studies including experiments with cancer cell lines have shown its activity as a tumor suppression gene in lung tumorigenesis and its implication in cell cycle progression and tumor growth [33,34].

Aberrant activation of the Wingless-related integration site (Wnt) signaling pathways plays an important role in the development of NSCLC [35]. Wnt inhibitory factor-1 (*WIF-1*) has been identified as an important Wnt antagonist which inhibits Wnt/β- catenin signaling by directly binding to Wnt proteins. Methylation of the *WIF-1* gene can lead to the loss of *WIF-1* expression which has been observed in numerous types of cancer including NSCLC [16,17,18,19,20]. Another important Wnt antagonist with tumor suppressor activity is the *APC* gene that is involved in cell migration and adhesion, transcriptional activation, and apoptosis; APC promoter methylation has a diagnostic role in NSCLC [36,37].

*BRMS1* is mostly studied in breast cancer for its role in the regulation of tumor metastasis [38]. Limited but significant evidence exists about its implication in NSCLC [39] and the association of its promoter methylation in plasma-cfDNA with poor progression free survival (PFS) [40]. *SLFN11* belongs to the Schlafen protein family, which has been implicated in cell proliferation, induction of immune responses, regulation of viral replication [41], and also in sensitizing cancer cells to DNA damaging agents [42]. In a small cohort of NSCLC patients who received platinum-based chemotherapy the presence of *SLFN11* hypermethylation was significantly associated with shorter PFS [43]. *SHISA3* was firstly reported as a novel tumor suppressor gene associated with tumorigenesis, invasion, and metastasis by promoting the degradation of β-catenin in lung cancer [44]. Lately, it was found that *SHISA3* ectopic expression in vivo combined with gefitinib or osimertinib treatment was negatively correlated with EGFR TKI resistance [45]. Promoter hypermethylation of *RARβ* has been suggested as a mechanism that regulates alveolar and epithelial differentiation and lung tumorigenesis. In NSCLC, *RARβ* hypermethylation may significantly contribute to the carcinogenesis and serve as a potential drug target and diagnostic marker [46]. *FOXA1* is one of the most significant transcription factors during epithelial-to-mesenchymal transition (EMT) and it is suggested that it may play an important role in the initiation of lung cancer metastasis [47]. *FOXA1* methylation was recently associated with the early detection of lung cancer [48] but it is not extensively studied in this type of cancer.

In the present study, we conducted for the first time a DNA methylation analysis of nine selected genes in plasma-cfDNA and paired CTCs of NSCLC patients before administration of osimertinib and at progression of disease. Our aim was to study for the first time whether DNA methylation for these gene-promoters is differentiated before administration of osimertinib and at progression of disease resistance and whether the presence of these epigenetic alterations at progression of disease could be a possible resistance mechanism.

## 2. Materials and Methods

**Figure 1:**
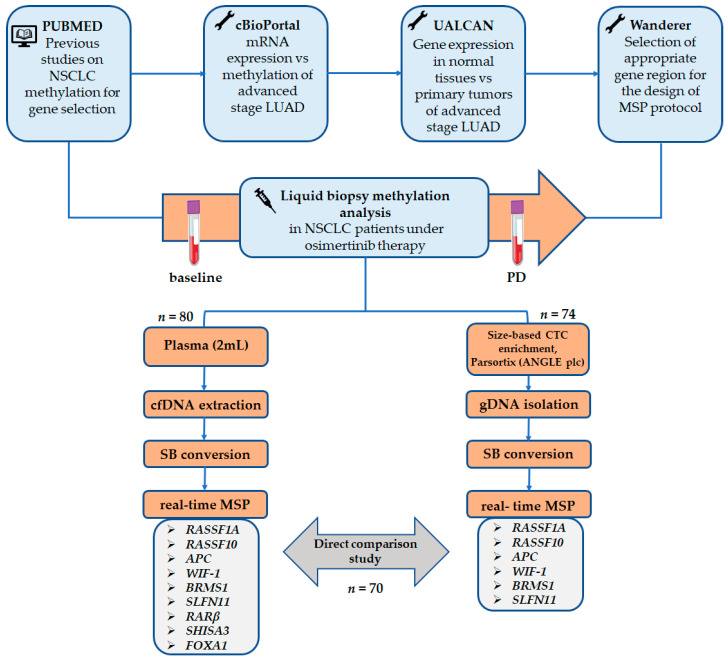
A schematic flowchart of the study (NSCLC: non-small cell lung cancer, LUAD: lung adenocarcinoma, MSP: methylation specific PCR, CTC: circulating tumor cells, SB: sodium bisulfite).

The flowchart of the study is shown in Figure 1.

### 2.1. TCGA Meta-Analysis

Firstly, a thorough search of literature was carried out to select cancer related genes where their DNA methylation was implicated NSCLC or that would serve as tumor suppressor genes. Then, a meta-analysis was performed based on data retrieved from The Cancer Genome Atlas (TCGA) Research Network through the online webtool cBioPortal (https://www.cbioportal.org/datasets (accessed on 15 July 2021)) to examine the correlation between mRNA expression and DNA methylation in lung adenocarcinoma samples for selected genes based on Pearson correlation analysis. Furthermore, we used the interactive web server UALCAN (http://ualcan.path.uab.edu (accessed on 15 July 2021)) for analyzing the gene expression levels of studied genes across normal tissues and cancerous tissue samples of stage III and IV lung adenocarcinoma patients based on TCGA level 3 RNA-Seq data.

### 2.2. Patients

Patients with histologically or cytologically documented *EGFR* mutated lung adenocarcinomas, previously progressed upon 1st and/or 2nd generation EGFR TKIs, were subsequently treated with osimertinib (AZD9291; Astra Zeneca, UK) in the context of a multicenter Phase II clinical study [ClinicalTrials.gov number: NCT02771314, registration date: 13 May 2016 and EudraCT number: 2016-001335-12, registration date: 13 April 2016] conducted by the Hellenic Oncology Research Group (HORG). All patients included in this clinical study had a Performance Status (ECOG) 0–1.

All patients and healthy donors (HD; *n* = 10) gave their written informed consent to participate in the study which has been approved by the National Drug Administration of Greece (EOF), the National Ethics Committee (35/00-03/16, 35/03-11/16) and the Institutional Ethical Committees of the HORG’s participating centers. The study was conducted in accordance with the Declaration of Helsinki.

In the present study, peripheral blood (PB) samples from 42 NSCLC patients, were subjected on plasma-cfDNA and CTC methylation analysis at two time points: (a) baseline: before treatment with osimertinib and (b) at the time of disease progression (PD). At the time of analysis, four patients were still under osimertinib therapy. Osimertinib was administered as a 2nd line treatment in 21/42 (50%) and as 3rd line in 21/42 (50%) upon their progression of disease with EGFR TKIs. The median age of patients was 66 (range: 43–87 years) and 31 (73.8%) of them were female.

### 2.3. Peripheral Blood Sampling and Processing

PB samples were obtained at two time points: (a) before the treatment initiation with osimertinib (baseline) and (b) at the time of disease progression (PD). At the time of analysis, four patients were still under osimertinib therapy. In total, 84 patient samples and 10 HD samples were further processed and analyzed following the same steps. PB (15 mL) was collected in tubes containing ethylenediaminetetraacetic acid (EDTA) as anticoagulant, after discarding the first 5mL of blood draw to avoid contamination of skin epithelial cells. Blood samples were centrifuged at 530× *g* for 10 min at room temperature (RT) and plasma was separated from buffy coat and erythrocytes. Plasma samples were then subjected to a second centrifugation at 16,000× *g* for 10 min at RT and transferred to a new tube. Aliquots of identical plasma samples from every single blood sampling were kept at −80 °C prior to cfDNA extraction. Buffy coat and erythrocytes were further processed for CTC enrichment as thoroughly described below.

### 2.4. Plasma-cfDNA Extraction

Purification of cfDNA was performed from 2 mL of plasma using the QIAamp Circulating Nucleic Acid Kit (Qiagen^®^, Hilden, Germany), according to the manufacturer’s instructions. It is a four-step protocol that combines the silica-based membrane extraction and purification of nucleic acids. The purified DNA maintains its methylation, allowing bisulfite conversion for downstream methylation analysis. The final elution volume of extracted cfDNA was 30 μL using Buffer AVE. In this study, 41 plasma-cfDNA baseline samples and 39 plasma-cfDNA samples at PD were available.

### 2.5. CTC Enrichment and Genomic DNA Extraction

After separating plasma from blood cells, an equal volume of removed plasma was replaced by adding phosphate-buffered saline (PBS, pH 7.3) into the cell pellet and then samples were proceeded for CTC enrichment in the size-based microfluidic device, Parsortix^™^ (ANGLE plc, Guildford, UK), using a 6.5 μm separation cassette, as previously described [49]. Captured cells were harvested in 200 μL of PBS. In a next step, genomic DNA (gDNA) was extracted from the CTC fraction using the TRIZOL-LS reagent (ThermoFisher Scientific, Waltham, MA, USA) and was dissolved in a final volume of 20 μL of 8 mmol/L NaOH, as previously described [49]. DNA concentration was measured in a NanoDrop-1000 spectrophotometer and calibrated with the recommended CF-1 standard solution. In this study, 39 CTC samples were collected at baseline and 35 CTC samples at PD.

### 2.6. Evaluation of DNA Integrity

After plasma-cfDNA and CTC derived-gDNA extraction, RT-qPCR with specific primers for amplifying a region in exon 20 wild type of the *PIK3CA* gene was performed, as previously described [50] to assess DNA integrity of all samples. Only samples that were positive for exon 20 *PIK3CA* amplification were further processed to sodium bisulfite (SB) treatment.

### 2.7. Sodium Bisulfite Treatment

After DNA extraction and quality control assessment, EZ DNA Methylation Gold Kit (ZYMO Research, Irvine, CA, USA) was used for SB treatment of plasma-cfDNA and CTC-derived gDNA samples, according to manufacturer’s instructions. All non-methylated cytosines were converted to uracil, while methylated cytosines remained unconverted. SB-treated DNA was stored at −80 °C until further use. Samples of distilled H_2_O and 100% methylated DNA were used as negative and positive control, respectively during every SB treatment procedure. SB treated samples were subsequently subjected to whole bisulfitome amplification (WBA) using a modified protocol of the EpiTect Whole Bisulfitome Kit (Qiagen^®^, Hilden, Germany). After SB-treatment, SB converted DNA integrity was assessed by a real-time methylation specific PCR (MSP) assay for *β-actin* (*ACTB*); only samples that were amplified were included in the study [51].

### 2.8. In Silico Primer and Probe Design

For all primers and probes that were de novo designed, we used Wanderer (http://maplab.imppc.org/wanderer/ (accessed on 21 July 2021), an interactive web browser for TCGA data, to identify all the significant methylated CGs of the promoter gene regions based on Illumina 450K analysis data in order to include as many as possible CGs in the in silico design. After selecting the appropriate gene region, Primer Premier 5 software (Premier Biosoft International, San Francisco, CA, USA) was used for the in silico design of primers and probes avoiding the formation of stable hairpin structures, primer dimers, cross dimers, and false priming sites. The specificity of the in silico designed sequences was validated by using the BLAST tool (https://blast.ncbi.nlm.nih.gov/Blast.cgi (accessed on 21 July 2021).

### 2.9. Real-Time Methylation Specific PCR

We used our previously developed and analytically validated real time MSP assays for RASSF1A [52], BRMS1 [25] and SLFN11 [53] genes. We further developed novel real-time MSP assays for RASSF10, APC, RARβ, FOXA1, WIF-1, and SHISA3 (Assay details are described in Supplementary File S1). All experiments for methylation analyses were performed in the cobas^®^ z 480 analyzer (Roche Molecular Systems, Inc., Pleasanton, CA, USA) in a total volume of 10 μL. Human placental genomic DNA (gDNA; Sigma-Aldrich, Burlington, MA, USA) was used as a real-time MSP negative control after SB-treatment, while Universal Methylated Human DNA Standard (ZYMO Research, Irvine, CA, USA) was used as fully methylated (100%) positive control. All MSP assays are qualitative and thus, we define a sample as positive for methylation when MSP amplification signal is present (Cq < 40.00) and as negative only in the complete absence of amplification signal.

### 2.10. Statistical Analysis

Statistical analysis was performed using SPSS Statistics 26.0 (IBM company, Armonk, NY, USA). The Pearson chi-square test was used to evaluate correlations between mRNA expression and DNA methylation. The chi-square test of independence was used to compare DNA methylation between different groups. The Mc Nemar test and Cohen’s kappa index were used to compare DNA methylation between the two time points. Survival distributions were estimated using the Kaplan-Meier method and compared across groups with the log-rank test. All statistical tests were two sided, and *p*-values less than 0.05 were considered statistically significant.

## 3. Results

### 3.1. TCGA Meta-Analysis of Methylated Genes in Lung Adenocarcinoma Tissue Samples

**Figure 2:**
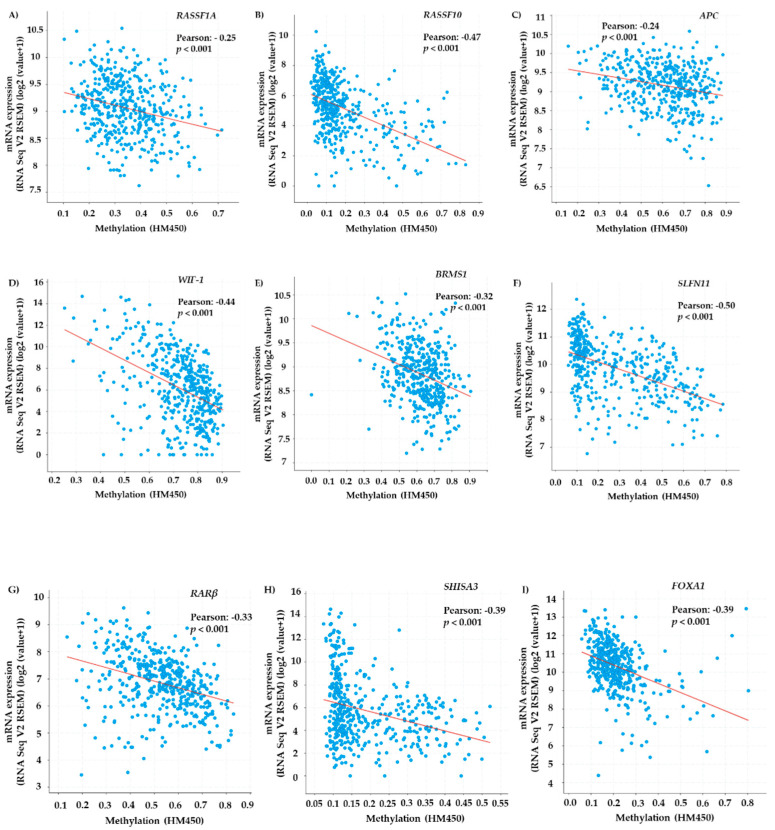
Association between mRNA expression and DNA methylation in lung adenocarcinoma tissues for (**A**) *RASSF1A*, (**B**) *RASSF10*, (**C**) *APC*, (**D**) *WIF-1*, (**E**) *BRMS1*, (**F**) *SLFN11*, (**G**) *RARβ*, (**H**) *SHISA3*, (**I**) *FOXA1* gene based on TCGA analysis within cBioPortal datasets.

Following the selection of genes based on published papers, TCGA meta-analysis was performed through cBioPortal platform (https://www.cbioportal.org/ (accessed on 15 July 2021) to explore correlations between DNA methylation and mRNA expression of selected genes. Data were retrieved by the Firehose Legacy study that included *n* = 584 lung adenocarcinoma tissue samples. The Pearson correlation analysis revealed that mRNA expression was negatively associated with DNA methylation in lung adenocarcinoma tissues for all genes indicating that DNA methylation of these genes may lead to their epigenetic silencing in lung adenocarcinoma (Figure 2).

**Figure 3:**
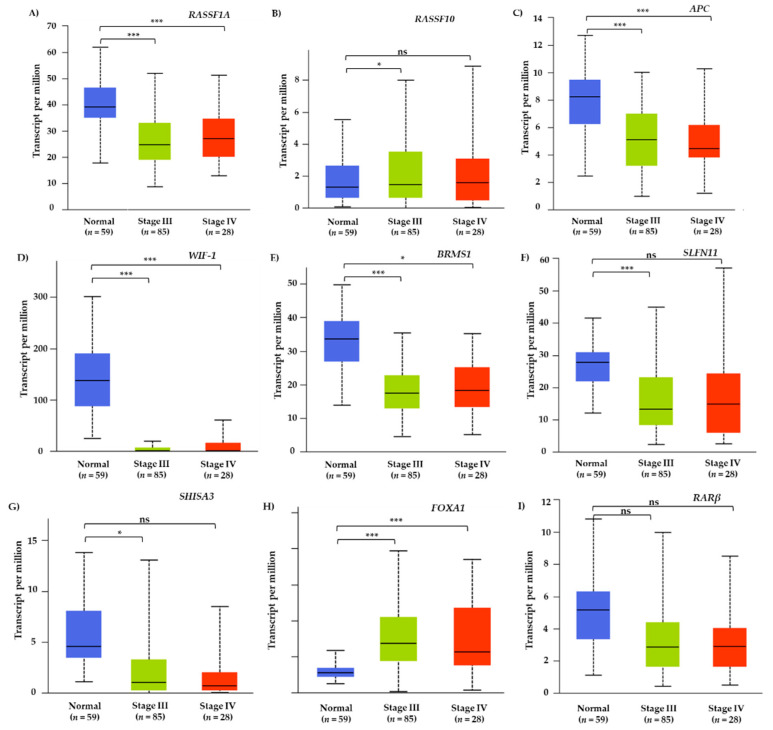
Gene expression levels between normal (*n* = 59) and stage III (*n* = 85) or stage IV (*n* = 28) LUAD tissue samples for (**A**) *RASSF1A*, (**B**) *RASSF10*, (**C**) *APC*, (**D**) *WIF-1*, (**E**) *BRMS1*, (**F**) *SLFN11*, (**G**) *RARβ*, (**H**) *SHISA3*, (**I**) *FOXA1* gene based on TCGA data from UALCAN web server (***: *p* < 0.001, * *p* < 0.05, ns: non-significant).

Furthermore, the UALCAN web server was used to explore differences in gene expression levels of these nine genes between normal (*n* = 59) and lung adenocarcinoma tissues. More specifically, TCGA level 3 RNA-Seq data were selected only for stage III (*n* = 85) and IV (*n* = 28) lung adenocarcinoma tissues to resemble the group of NSCLC patients that was further studied (Figure 3).

### 3.2. Plasma-cfDNA Methylation Analysis

All HDs plasma-cfDNA samples (*n* = 10) were analyzed in the same way for the methylation of these nine genes; no methylation was detected in any of the plasma-cfDNA samples. The methylation status of patients’ plasma-cfDNA samples is described in detail below:

- *RASSF1A:* Before treatment with osimertinib, methylation of *RASSF1A* was detected in 3/41 (7.3%) patients, whereas at PD it was observed in 7/39 (17.9%) patients. One patient (pt#38) maintained *RASSF1A* methylation at both time points, whereas pt#44 and pt#46 were negative at PD.
- *RASSF10:* Methylation for *RASSF10* was not detected at any time point.
- *APC:* 5/41 (12.2%) patients were positive for *APC* methylation during baseline and three of these five (pt#1, pt#18, pt#20) maintained methylation also at PD. In total, 9/39 (23.1%) patients were detected with *APC* methylation at PD.
- *WIF-1: WIF-1* methylation was detected in 2/41 (4.9%) patients at baseline and in 6/39 (15.4%) patients at PD.
- *BRMS1:* Before osimertinib treatment *BRMS1* methylation was detected in only one patient (pt#28) who was also positive at PD. At PD, 5/39 (12.8%) patients were positive for *BRMS1* methylation.
- *SLFN11:* Only one patient was detected with *SLFN11* methylation at baseline while 3/39 (7.7%) patients were positive at PD.
- *RARβ:* Before treatment with osimertinib, no methylation was detected at all whereas 2/39 (5.1%) patients were positive at PD.
- *SHISA3:* SHISA3 methylation was detected in only one patient (pt#17) at baseline and in only one (pt#38) at PD.
- *FOXA1:* 2/41(4.9%) patients were detected positive for *FOXA1* methylation at baseline whereas 3/39 (7.7%) patients at PD. Only one patient (pt#38) was positive for *FOXA1* methylation at both time points.

**Figure 4:**
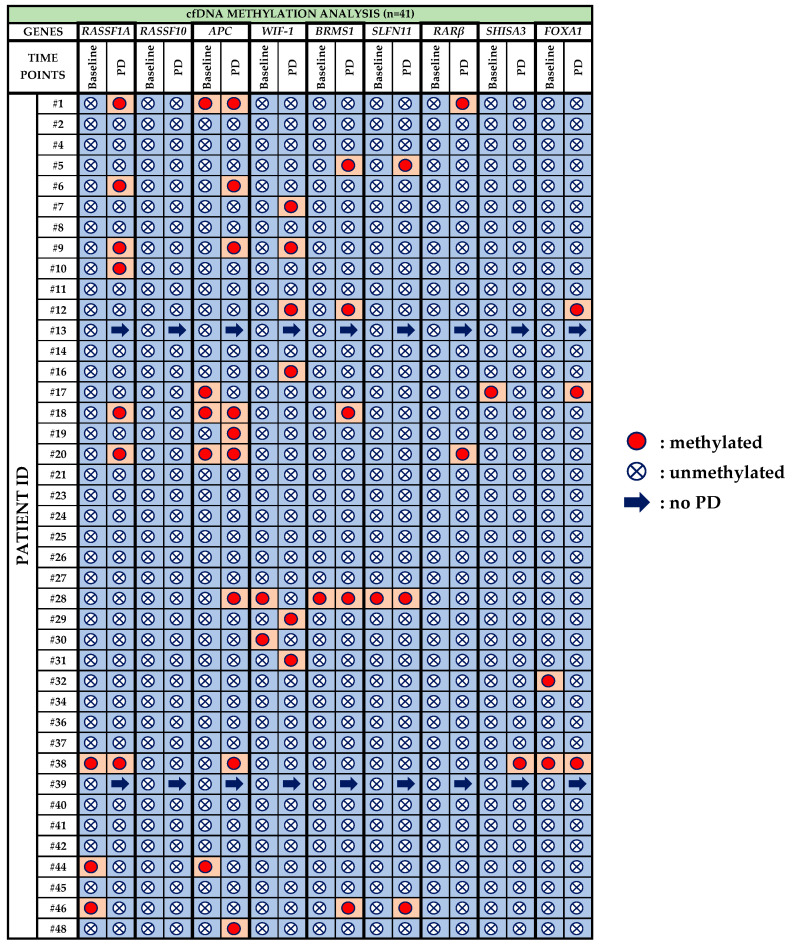
Methylation status of nine selected genes in plasma-cfDNA of NSCLC patients before osimertinib initiation (*n* = 41) and at progression of disease (*n* = 39).

Methylation rates for *RASSF1A*, *APC*, *WIF-1, FOXA1*, *RARβ*, *BRMS1* and *SLFN11* were higher at PD compared to that at baseline but without any statistically significant differences. Furthermore, patients found positive for at least one methylation marker were 10/41 (24.4%) at baseline and 17/39 (43.6%) at PD. Interestingly, among those patients carrying more than one methylation marker, the presence of *RASSF1A* methylation was strongly correlated with the *APC* methylation (Fisher’s exact test, *p* < 0.001, k = 0.687). All results on the methylation status of these nine genes in plasma-cfDNA are summarized in Figure 4.

### 3.3. CTC Methylation Analysis

**Figure 5:**
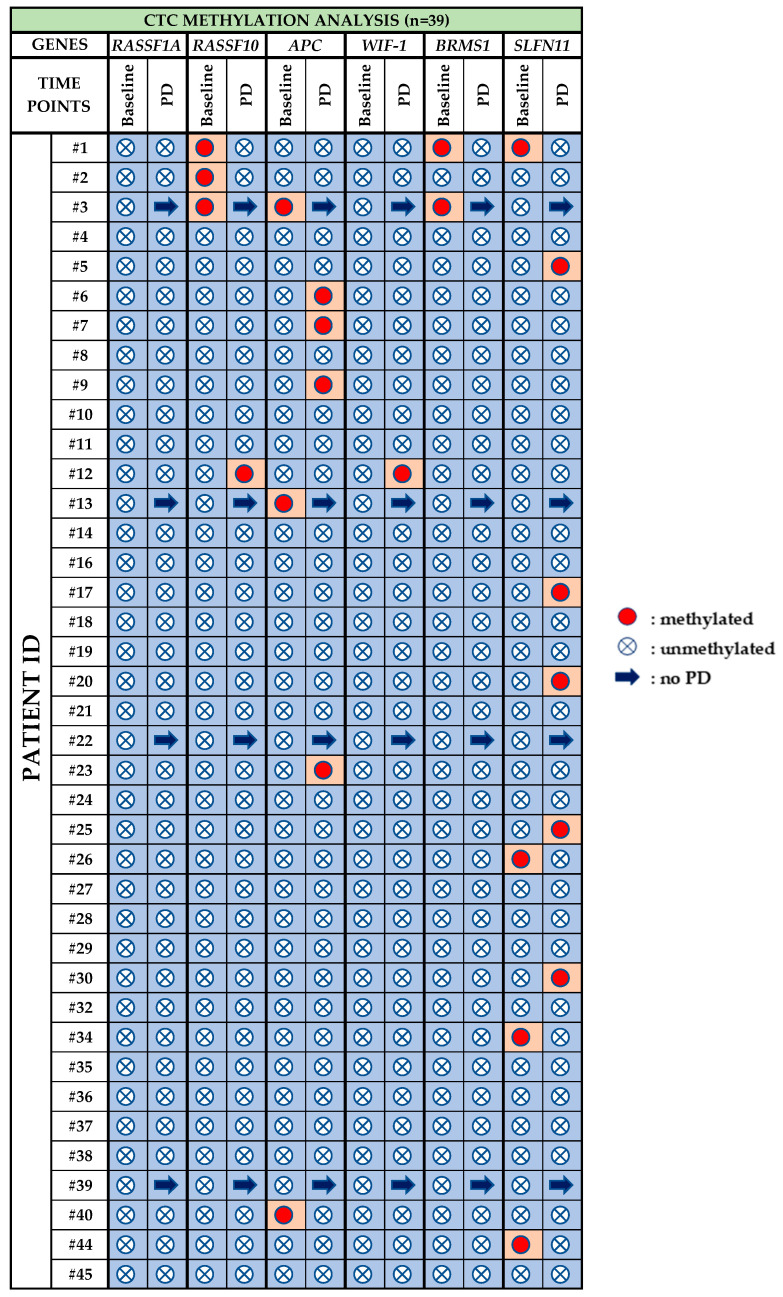
Methylation status of six selected genes in CTCs of NSCLC patients before osimertinib initiation (*n* = 39) and at progression of disease (*n* = 35).

Peripheral blood of 10 HDs was analyzed in exactly the same way as for patient samples for CTC. Methylation was not detected for any of these genes in HDs samples. Figure 5 demonstrates the methylation status of the six genes in CTCs of NSCLC patients under osimertinib therapy. More specifically:

- *RASSF1A:* Methylation for *RASSF1A* was not detected at any time point.
- *RASSF10:* Before treatment with osimertinib, methylation of *RASSF10* was detected in 3/39 (7.3%) patients, whereas at PD it was observed in 1/35 (2.8%) patient.
- *APC:* 3/39 (7.7%) patients were positive for *APC* methylation at baseline, while at PD, 4/35 (11.4%) were positive for *APC* methylation.
- *WIF-1: WIF-1* methylation was not detected at baseline whereas at PD only one sample (pt#12) was found positive.
- *BRMS1:* Before osimertinib treatment *BRMS1* methylation was detected in 2/39 (5.1%) patients, while at PD, no *BRMS1* methylation was detected.
- *SLFN11:* At baseline, *SLFN11* methylation was detected in 4/39 (10.2%) patients that were found negative at PD. At PD 5/35 (14.3%) patients were found positive for *SLFN11* methylation.

Overall, the methylation rates for *APC*, *WIF-1* and *SLFN11* were increased at PD, while the methylation rates for *RASSF10* and *BRMS1* were decreased but without any statistical significance. At baseline, 7/39 (17.9%) samples were found positive for at least one methylation marker, whereas at PD 10/35 (28.6%) were found positive for at least one methylation marker.

### 3.4. Direct Comparison of the Methylation Status of Six Genes between Plasma-cfDNA and Paired CTCs

**Figure 6:**
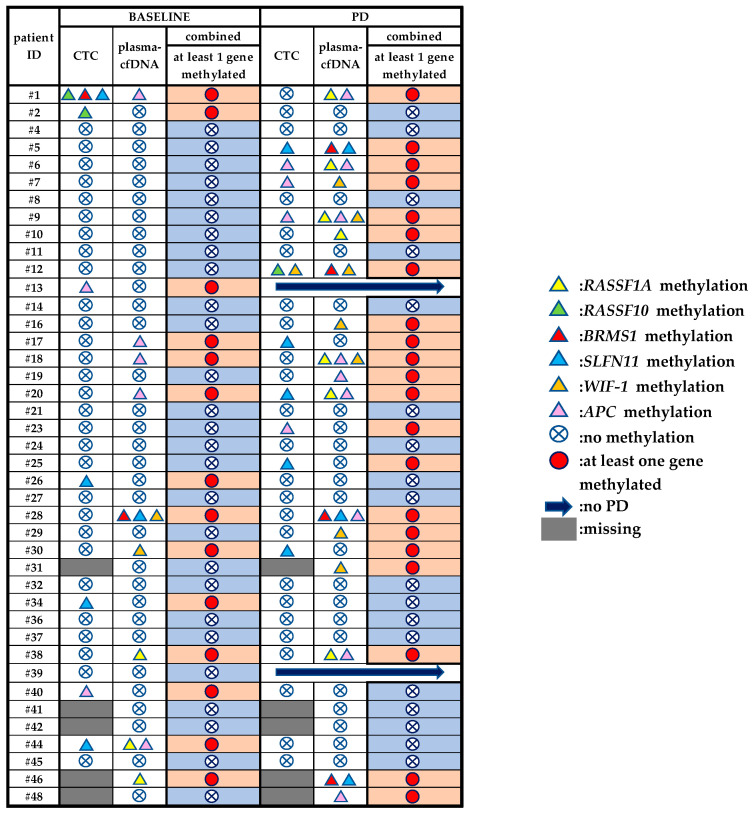
Direct comparison of DNA methylation markers in plasma-cfDNA and paired CTCs before osimertinib and at PD.

We evaluated in total 70 matched plasma-cfDNA and CTC samples, 36 at baseline and 34 at PD for the methylation of six genes (*RASSF1A*, *RASSF10*, *APC*, *WIF-1*, *BRMS1*, *SLFN11*). At baseline, there was no agreement between plasma-cfDNA and CTCs in terms of methylation, whereas at PD there was an agreement in patient #5 for *SLFN11*, patients #6 and #9 for *APC* and patient #12 for *WIF-1*. Totally different methylation patterns were observed between plasma-cfDNA and CTC matched samples for patients #1 and #44 at baseline and for patients #7 and #20 at PD. Moreover, as can be seen in Table 1, there were patients’ samples positive for various methylation markers solely in CTCs (Pt#2, Pt#13, Pt#17, Pt#23, Pt#25, Pt#26, Pt#30, Pt#34, Pt#40) and others solely in plasma-cfDNA (Pt#1, Pt#10, Pt#16, Pt#20, Pt#28, Pt#30, Pt#38) either at baseline or at PD. All results are shown in Figure 6.

The presence of CTCs in identical blood draws was already verified through our previous studies by (a) triple immunofluorescence (IF) for *CK* (pan cytokeratin Ab, CK-8, CK-18, CK-19) and/or *VIM*, (b) RT-qPCR for the detection of *CK-8, CK-18, CK-19*, and *VIM* [49] and (c) crystal digital PCR (cdPCR) for the detection of *EGFR* mutations in CTCs [54].

### 3.5. DNA Methylation Analysis of Plasma-cfDNA and Paired CTCs

We have also evaluated the presence of at least one methylation marker both in plasma-cfDNA and paired CTCs at both time points (Figure 6). At baseline, 14/41 (34.1%) patients were found positive for at least one methylated marker, whereas 21/39 (53.8%) patients were positive at PD. More specifically, in 8/41 (19.5%) patients, methylation was detected at both time points, whereas in 13/41 (31.7%) patients, methylation was not detected at all. Among those patients that progressed, methylation in at least one methylation marker was detected only at PD in 13/39 (33.3%) patients. By comparing these two time points of osimertinib therapy, a significant increase in methylation for at least one marker was observed at PD in contrast to baseline (McNemar test, *p* = 0.031).

We combined the information on DNA methylation status in plasma-cfDNA and CTC at PD, and divided NSCLC patients into two groups: Group A included patients who progressed within 13 months and Group B included those who progressed after 13 months or did not progress at all at the time of analysis and were still under osimertinib. As shown in Table 1, 18 out of 19 (94.7%) of NSCLC patients for whom at least one of the genes studied was methylated in cfDNA and/or CTC at PD progressed much earlier (within 13 months) whereas only one out of 19 (1.2%) of these DNA methylation positive NSCLC patients progressed later than 13 months. Among the 41 NSCLC patients studied, 32/41 (78%) relapsed earlier than 13 months and only 9/41 (21.9%) after.

**Figure 7:**
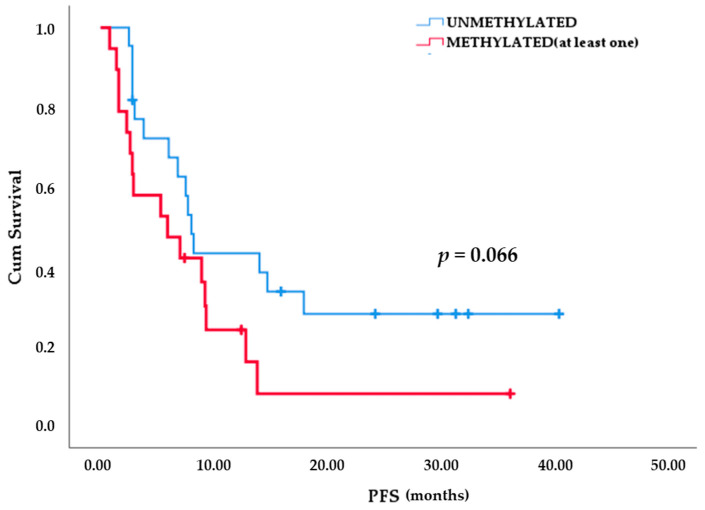
Kaplan-Meier analysis based on the methylation status for at least one marker in cfDNA and/or CTC at PD revealed a slight difference in terms of PFS.

Kaplan-Meier analysis indicated differences in time-to-progression between NSCLC patients that were found methylated for at least one marker at PD in cfDNA and/or CTC and patients that were found negative for DNA methylation of these markers. More specifically patients who were positive for at least one methylated marker tended to progress earlier than those who were negative for DNA methylation for all markers (8.5 vs. 16.7 months, *p* = 0.066; Figure 7).

## 4. Discussion

In the present study, we directly compared DNA methylation of nine selected genes in CTCs and paired plasma-cfDNA of NSCLC patients that are longitudinally monitored during osimertinib therapy, to evaluate a potential role in NSCLC progression and EGFR TKI therapy resistance. Our results were derived by highly specific and highly sensitive analytically validated real time MSP molecular assays, following strict preanalytical and analytical quality control steps.

As it is widely known, the subclonal emergence of EGFR mutation T790M is a common event induced during treatment in EGFR mutant NSCLC [55,56]. Parallel or downstream signaling pathways are dysregulated by epigenetic mechanisms in cancer [57,58], and adaptive treatment resistance through epigenetic modifications that gradually lead to the dominance of drug-resistant cells is an alternative process of treatment failure [16]. The concept of the drug persistent cells that are potent enough to escape treatment effect through epigenetic mechanisms has also been demonstrated at a single cell level [59,60].

In our study, prior to liquid biopsy analysis, a meta-analysis of lung adenocarcinoma TCGA data revealed that all nine genes selected presented low or moderate though significant negative correlation between mRNA expression and DNA methylation levels. Moreover, the UALCAN webtool was used to explore the differences in gene expression levels between normal tissues and cancerous tissue samples of advanced stage lung adenocarcinoma patients (stage III and IV) and further confirm their possible function as tumor suppressor genes. This meta-analysis revealed that almost in all cases, lung adenocarcinoma tissue samples presented lower gene expression levels indicating the possible effect of DNA methylation of these genes on their transcriptional silencing. Our aim was to exploit the maximum information that TCGA data provide by using the online webtools and software; however, there were no available studies including data for NSCLC patients under osimertinib therapy and as a result we only used data concerning a broader category of lung adenocarcinoma patients’ samples. Following the TCGA meta-analysis, we analyzed liquid biopsy samples of NSCLC patients under osimertinib therapy before treatment initiation and at PD for DNA methylation of these nine selected genes.

To the best of our knowledge there is no previous study on the direct comparison of plasma-cfDNA and CTC methylation profiling in NSCLC during osimertinib therapy. Plasma-cfDNA methylation analysis revealed a heterogeneous profile of DNA methylation for these nine markers among NSCLC patients and an increase of DNA methylation rates in total for ‘at least one marker’ at PD. Methylation of *RASSF1A*, *APC*, and *WIF-1* was detected at higher percentages at PD in plasma-cfDNA samples. As per CTC methylation analysis a completely different profile was revealed compared to the plasma-cfDNA methylation analysis performed in the same blood draws. Overall, methylation was detected in lower rates in CTCs than in plasma-cfDNA but there was again an increase at PD against baseline samples. Direct comparison of DNA methylation of six of these genes (*RASSF1A*, *RASSF10*, *APC*, *WIF-1*, *BRMS1*, and *SLFN11*) between plasma-cfDNA and CTCs at baseline and at PD revealed different methylation patterns indicating that CTC and plasma-cfDNA analysis give complementary information and a high level of heterogeneity between patients even at the DNA methylation level.

Limited but significant scientific evidence attests epigenetic modifications that negatively affect EGFR TKI treatment outcome and that their combination with epigenetic drugs could be very promising. One of the earlier studies based on PC-9 cancer cell line have shown that PTEN hypermethylation conferred resistance to gefitinib and erlotinib that was restored with 5-aza-2′-deoxycytidine (5AZA-CdR) administration [61]. Activation of Wnt signaling, observed in tumor samples through the methylation of *SFRP5* gene, could cause tumor resistance to EGFR TKIs and, on account of this it was associated with poorer treatment outcomes [62]. The cooperative effects of histone deacetylase inhibitors (HDACis) and EGFR TKIs in vitro displayed synergistic effects on H3K4 methylation and increased response to therapy [63]. Another effective combination was the administration of the DNA methyl transferase inhibitor, decitabine along with gefitinib in PC9/GR cells through demethylation of RASSF1A and GADD45β [64]. A less common methylation-associated mechanism was found to be involved in the generation of T790M mutation that was induced by the activity of cytidine deaminase (AICDA) expression [65]. Very recently, a genome-wide DNA methylation analysis revealed that HOXB9 DNA methylation was linked to intrinsic EGFR-TKI resistance and complementary to mutation profiling could discern whether lung adenocarcinoma patients would benefit from EGFR-TKI treatment [66]. However, all these studies were performed in lung cancer cell lines or primary tissues and have not thus compared DNA methylation status before and after treatment. According to our study, there was a trend for a statistically important difference in PFS between patients who were positive for DNA methylation of at least one marker at PD and those patients that were negative for DNA methylation of at least one of these markers (8.5 vs. 16.7 months, *p* = 0.066).

What really matters during longitudinal monitoring of patients is the early prediction of disease progression; both can be achieved through liquid biopsy analysis. However, there are only a few studies so far involving liquid biopsy analysis for the elucidation of therapeutic resistance to TKI treatment. A recent study, that was based on only eight patients, and combined a methylation ratio model with somatic mutation profiling in cfDNA predicted resistance to Osimertinib prior to morphological PD [67]. In a previously published genome-wide methylation analysis, it was shown that different resistance mutation profiles in cfDNA were associated with different methylation changes highlighting the heterogeneity of acquired resistance to EGFR TKIs and the complex interrelationship between DNA methylation and genetic alterations [68].

## 5. Conclusions

In the present study, we conducted for the first time a DNA methylation analysis of nine selected genes in plasma-cfDNA and paired CTCs of NSCLC patients before the administration of osimertinib and at progression of disease and explored whether the presence of epigenetic alterations at progression of disease could be a possible resistance mechanism. According to our plasma-cfDNA analysis, DNA methylation in these genes was detected at higher percentages at PD. Combination of DNA methylation results in CTC and plasma-cfDNA indicated that the methylation rates for all genes tested were significantly elevated at progression of disease after osimertinib treatment when compared to baseline, indicating that DNA methylation of these genes may be associated with epigenetic resistance during osimertinib therapy. Elucidation of DNA methylation profiles in liquid biopsy still remains an unexplored but promising field with great potential. Our study evaluated for the first time whether DNA methylation of specific gene promoters detected in plasma cfDNA and/or CTCs was associated with resistance to osimertinib. Future prospective studies including larger cohorts of patients are needed to confirm our findings and investigate whether EGFR mutant NSCLC patients could benefit from subsequent or combinatorial epigenetic therapy.

## Figures and Tables

**Table 1:** DNA methylation status in cfDNA and/or CTCs in respect to time-to-progression.

Methylation Status	Early PD (Up to 13 Months)	Late PD (More Than 13 Months)	Total
Unmethylated	14	8	22
Methylated	18	1	19
(at least one gene)			
Total	32	9	41

## Data Availability

The data presented in this study are available on request from the corresponding author. The data are not publicly available due to ethical restrictions.

## Supplementary Materials

The following are available online at https://www.mdpi.com/article/10.3390/cancers13235974/s1, File S1: Real-time MSP Protocols.
