# Supplementary material for: DNA Methylation Analysis in Plasma Cell-Free DNA and Paired CTCs of NSCLC Patients before and after Osimertinib Treatment
Source: Cancers (Basel). 2021 Nov 27;13(23):5974. doi: 10.3390/cancers13235974 (PMC8656722; doi:10.3390/cancers13235974)
Supplement: Supplementary file 1 [file cancers-13-05974-s001.zip › cancers-1451801-supplementary.pdf]

cancers

MDPI

Article

DNA Methylation Analysis in Plasma Cell-Free DNA and Paired CTCs of NSCLC Patients before and after Osimertinib Treatment

Aliki Ntzifa [1], Dora Londra [1], Theodoros Rampias [2], Athanasios Kotsakis [3], Vassilis Georgoulias [4] and Evi Lianidou [1,*]

[1] Analysis of Circulating Tumor Cells Lab, Lab of Analytical Chemistry, Department of Chemistry, National and Kapodistrian University of Athens, 15771 Athens, Greece; alntzi@chem.uoa.gr (A.N.); doralo@chem.uoa.gr (D.L.)
[2] Basic Research Center, Biomedical Research Foundation of the Academy of Athens, 11527 Athens, Greece; trampias@bioacademy.gr
[3] Faculty of Medicine, School of Health Sciences, University of Thessaly, 41110 Larissa, Thessaly, Greece; thankotsakis@uth.gr
[4] Department of Medical Oncology, Hellenic Oncology Research Group (HORG), 11471 Athens, Greece; georgulv@otenet.gr
* Correspondence: lianidou@chem.uoa.gr; Tel.: +30-210-727-4311

Citation: Ntzifa, A.; Londra, D.; Rampias, T.; Kotsakis, A.; Georgou-lias, V.; Lianidou, E. DNA Methyla-tion Analysis in Plasma Cell-Free DNA and Paired CTCs of NSCLC Patients before and after Osimertinib Treatment. *Cancers* **2021**, *13*, 5974. https://doi.org/10.3390/cancers13235974

Academic Editor: David Wong

Received: 21 October 2021
Accepted: 25 November 2021
Published: 27 November 2021

Publisher's Note: MDPI stays neu-tral with regard to jurisdictional claims in published maps and institu-tional

Copyright: © 2021 by the authors. Licensee MDPI, Basel, Switzerland. This article is an open access article distributed under the terms and conditions of the Creative Commons Attribution (CC BY) license (http://creativecommons.org/licenses/by/4.0/).

Supplementary

Real-time MSP Protocols

APC

FORWARD: 5′- TTATTGCGGAGTGCGGGT -3′
REVERSE: 5′- CGAACTCCCGACGAAAATAAA -3′

The PCR reaction mix consisted of 1X PCR buffer (Promega, USA), 2.0 mM $MgCl_2$ (Promega, USA), 0.15 μM of each dNTP (Invitrogen, USA), 0.15μg/μL BSA (Sigma, Germany), 0.20 μM of each primer (Integrated DNA Technologies, USA), 1X LC Green® (Idaho Technology, USA) and 0.05 U/μL GoTaq® DNA polymerase (Promega, USA). dH_2O was added to a final volume of 10μL. Protocol conditions were: 1 cycle at 95°C for 2min, followed by 45 cycles of: 95°C for 10s, 66°C for 15s and 72°C for 20s, and a final cooling cycle at 40°C for 30s.

RASSF10

FORWARD: 5′- CGTCGTTTTAGTAGATTTCGGTC -3′
REVERSE: 5′- CGTCGAAACAAATAATACGACG -3′

The PCR reaction mix consisted of 1X PCR buffer (Promega, USA), 2mM $MgCl_2$ (Promega, USA), 0.2 μM of each dNTP (Invitrogen, USA), 0.25μg/μL BSA (Sigma, Germany), 0.25 μM of each primer (Integrated DNA Technologies, USA), 1X LC Green® (Idaho Technology, USA) and 0.05 U/μL GoTaq® DNA polymerase (Promega, USA). dH_2O was added to a final volume of 10μL. Protocol conditions were: 1 cycle at 95°C for 2min, followed by 45 cycles of: 95°C for 10s, 65°C for 20s and 72°C for 20s, and a final cooling cycle at 40°C for 30s.

SHISA3

FORWARD: 5′- TAGTTTTAGAGGCGCGATTC -3′
REVERSE: 5′- CAAATACAAACGACCGCG -3′

The PCR reaction mix consisted of 1X PCR buffer (Promega, USA), 2.5 mM $MgCl_2$ (Promega, USA), 0.2 μM of each dNTP (Invitrogen, USA), 0.25μg/μL BSA (Sigma, Germany), 0.25 μM of each primer (Integrated DNA Technologies, USA), 1X LC Green® (Idaho Technology, USA) and 0.05 U/μL GoTaq® DNA polymerase (Promega,

USA). dH_2O was added to a final volume of 10µL. Protocol conditions were: 1 cycle at 95°C for 2min, followed by 45 cycles of: 95°C for 10s, 63°C for 20s and 72°C for 20s, and a final cooling cycle at 40°C for 30s.

FOXA1

FORWARD: 5′- GGCGGCGACGTTAAGAC -3′
REVERSE: 5′- CGCTCAACGTAAACATCTTACT -3′

The PCR reaction mix consisted of 1X PCR buffer (Promega, USA), 2.0 mM $MgCl_2$ (Promega, USA), 0.15 µM of each dNTP (Invitrogen, USA), 0.15µg/µL BSA (Sigma, Germany), 0.20 µM of each primer (Integrated DNA Technologies, USA), 1X LC Green® (Idaho Technology, USA) and 0.05 U/µL GoTaq® DNA polymerase (Promega, USA). dH_2O was added to a final volume of 10µL. Protocol conditions were: 1 cycle at 95°C for 2min, followed by 45 cycles of: 95°C for 10s, 66°C for 20s and 72°C for 20s, and a final cooling cycle at 40°C for 30s.

RARβ

FORWARD: 5′- CGAGAACGCGAGCGATTC -3′
REVERSE: 5′- TCGACCAATCCAACCGAAAC -3′

The PCR reaction mix consisted of 1X PCR buffer (Promega, USA), 2.0 mM $MgCl_2$ (Promega, USA), 0.15 µM of each dNTP (Invitrogen, USA), 0.15µg/µL BSA (Sigma, Germany), 0.20 µM of each primer (Integrated DNA Technologies, USA), 1X LC Green® (Idaho Technology, USA) and 0.05 U/µL GoTaq® DNA polymerase (Promega, USA). dH_2O was added to a final volume of 10µL. Protocol conditions were: 1 cycle at 95°C for 2min, followed by 45 cycles of: 95°C for 10s, 65°C for 15s and 72°C for 20s, and a final cooling cycle at 40°C for 30s.

WIF-1

FORWARD: 5′- TTATACGTTTATTTCGCGGGC -3′
REVERSE: 5′- GACCGCCACTTAAAAACGCT -3′
PROBE: 56-FAM/ ATTGT+GAATGTAGTT +TCGGGGGTT/3BHQ_1

The PCR reaction mix consisted of 1X PCR buffer (Promega, USA), 3.0 mM $MgCl_2$ (Promega, USA), 0.15 µM of each dNTP (Invitrogen, USA), 0.15µg/µL BSA (Sigma, Germany), 0.20 µM of each primer (Integrated DNA Technologies, USA), 0.30 µM of LNA probe (Integrated DNA Technologies, USA) and 0.05 U/µL GoTaq® DNA polymerase (Promega, USA). dH_2O was added to a final volume of 10µL. Protocol conditions were: 1 cycle at 95°C for 2min, followed by 45 cycles of: 95°C for 10s, 58°C for 15s and 72°C for 20s, and a final cooling cycle at 40°C for 30s.
